# The Potential Effect of Novel Coronavirus SARS-CoV-2 on NK Cells; A Perspective on Potential Therapeutic Interventions

**DOI:** 10.3389/fimmu.2020.01692

**Published:** 2020-07-10

**Authors:** Anahid Jewett

**Affiliations:** ^1^Division of Oral Biology and Medicine, The Jane and Jerry Weintraub Center for Reconstructive Biotechnology, UCLA, Los Angeles, CA, United States; ^2^The Jonsson Comprehensive Cancer Center, UCLA School of Dentistry and Medicine, Los Angeles, CA, United States

**Keywords:** COVID-19, NK cells, virus, immune cell, SARS-CoV-2

## Abstract

Coronavirus-induced disease-2019 (COVID-19) continues to cause significant morbidity and mortality worldwide. While studies on SARS-CoV-2 effects on immune cell function continue to progress, we know very little about the significance of depletion of key immune effectors by the virus in the mortality and morbidity of the disease. This commentary outlines what is the reported literature thus far on the effect of virus on NK cells known to kill virally infected cells. It also underscores the necessity for the future comprehensive studies of NK cells in SARS-CoV-2 infected individuals and animal models to better understand the role and significance of reported NK cell depletion and functional inactivation in disease morbidity and mortality, in hope to design effective therapeutic interventions for the disease.

Coronavirus-induced disease-2019 (COVID-19) poses a great public health threat, and presents a complex challenge for epidemiologists and public health professionals around the planet, as the disease has shifted from a regional epidemic to a worldwide pandemic in a short period of time. The toll that the disease has had on the global level continues to increase as the virus reaches all continents, except Antarctica, afflicting more than 180 countries. Initial reports of COVID-19 disease came from Wuhan, China in late December 2019, as patients began complaining about unexplained respiratory infections, which later was coined as “pneumonia of unknown etiology” (1). Shortly after surfacing of the virus several independent laboratories identified the causative agent of COVID-19 disease, ultimately naming it as severe acute respiratory syndrome coronavirus 2 (SARS-CoV-2) (2, 3).

While the search is continuing to uncover the infectious path of SARS-CoV-2, several key findings have led the infectious disease experts to partly uncover the mechanisms of the original spread to humans. By phylogenetically comparing SARS-CoV-2 to other coronaviruses, it was noted that the new virus was highly identical to other coronaviruses that had originated from bats (3, 4). However, to date the complete transmission route remains elusive.

Despite the novelty of this particular strain of coronavirus, the SARS-CoV-2 is not without precedent. Outbreaks in the past decades, such as severe acute respiratory syndrome (SARS) and Middle East respiratory syndrome (MERS), identified viruses that fall into the same category of coronaviruses, which are single-stranded RNA viruses (+ssRNA) that morphologically have been determined to express crown-like spikes on their surfaces. However, the difference seen between prior species of coronaviruses and SARS-CoV-2 partly lies in their respective symptom presentations in patients. Compared to SARS and MERS, the symptoms of COVID-19 disease are not presented earlier in the infectious cycle, which may be a reason for the greater ability of viral transmission in patients (4). The incubation period of the SARS-CoV-2 is relatively longer than those of SARS and MERs (7–14 days vs. 5.0–6.9 and 4.4–6.9, respectively) (4). In addition to its longer incubation period, the mean reproductive number (R_0_) of SARS-CoV-2 has also been estimated to range from 2.20 to 3.58, indicating that each infected patient can on average transmit the disease to two to three other individuals (5, 6).

According to the available COVID-19 clinical data, most patients fall into the range of 30–79 years of age, although several cases have been identified in younger individuals and in children recently (7). For infected patients, severity of symptoms has been classified as mild, severe, and critical. This spectrum of disease widely varies, as clinical presentation in infected individuals have ranged from asymptomatic infection to severe respiratory failure (2). Asymptomatic transmission of SARS-CoV-2 poses a great public health challenge in containment efforts, as previous reports have noted as much as 12.6% of case reports to be pre-symptomatic transmission (8). However, the main characteristic symptoms of COVID-19 disease have included fevers, fatigue, dry cough and respiratory distress.

The number of SARS-CoV-2 infected cases will certainly continue to rise worldwide especially now that many countries have chosen to relax the rules of social distancing and isolation due to the reopening of the economy and the work force. One of the most troubling factors about this disease is the lack of adequate understanding of the virus and the mechanisms by which it mediates the underlying pathology in humans. The problem has been compounded by the limited ability of the research laboratories to conduct studies due to the implementation of social distancing since many academic university laboratories have either been shut down or been operating at a minimum capacity. Although the existing novel therapeutic strategies and research on potential vaccines are important directions, they will not be sufficient to provide adequate progress to fully understand the potential of the virus to infect individuals and the underlying mechanisms by which the virus causes pathology. Containment efforts, through quarantines and social distancing, hand washing and wearing mask are important directions to mitigate the spread of SARS-CoV-2 infections. However, at the moment, we do not have the capability of large scale testing which would be necessary for the identification and isolation of asymptomatic and symptomatic patients to halt the chain of viral transmission. Therefore, until the existing public health measures are able to curtail the transmission and bring the disease somewhat under control, the research laboratories will not be able to fully engage in the studies of COVID-19 disease worldwide, thereby slowing the discoveries of more effective treatments and vaccines.

Progression models of COVID-19 disease paint a dismal forecast for the duration of the outbreak, and therefore, warrant the discovery of novel treatments to alleviate disease symptoms that will supplement containment efforts. Researchers continue to search for viable treatments to alleviate symptoms of the disease and to eradicate the spread of the disease. The search for effective treatments for COVID-19 disease has featured studies advocating for the potential use of the anti-malarial drugs, hydroxychloroquine. The placebo controlled studies of hydroxychloroquine have not shown any efficacy, and indeed, in certain cases they have exhibited dangerous side effects in patients. Others have advocated the immunologic path in support of monoclonal antibody therapy (9, 10). On the other hand Remdesivir, an antiviral drug, was shown to be superior to placebo in shortening the time to recovery in adults hospitalized with Covid-19 disease with the evidence of lower respiratory tract infection (11). The development of a SARS-CoV-2 vaccine would utilize the adaptive immune system to combat symptoms in patients, along with providing a preventative measure for healthy individuals. However, current timelines estimate that vaccine development could take up to anywhere from 12 to 18 months.

While studies on SARS-CoV-2's effects on immune functions continue to progress, published studies concerning other coronaviruses may shed some light on how the immune system may be employed to mitigate COVID-19 disease. Based on experiences with coronaviruses in SARS and MERS, it has been suggested that the SARS-CoV-2 infection may also trigger major immunological changes, such as delayed or suppressed Type 1 IFN response and the influx of activated neutrophils and inflammatory monocytes/macrophages (12). In addition, the effect of virus on host following infection has demonstrated severe changes in the proportion of different immune effectors (13). In particular, in the peripheral blood of patients that were infected with SARS, it was noted that there were significantly lower numbers of natural killer (NK) cells compared to healthy subjects (14). Such a profile has also been extended to the immune responses of COVID-19 individuals. A study of 452 COVID-19 individuals demonstrated that the numbers of NK, B and T cells were significantly decreased, with more severe cases being associated with greater decreases in the numbers (15), Please see the references (16–19) for reviews on the potential mechanisms of inactivation and loss of NK cells.

Upon admission, the neutrophil counts were remarkably higher in patients with severe COVID-19 disease than in the mild cases, whereas the total lymphocyte counts were significantly lower in severe cases when compared to the mild cases (15). The numbers of NK cells and total T cells as well as CD8+ T cells were significantly lower in patients exhibiting severe symptoms when compared to those with the mild symptoms and healthy controls (20). Thus, a correlation could be seen with severe decrease in NK and T cell numbers and the extent of severity of the disease. Furthermore, the function of NK and CD8+ T cells was found to be suppressed along with the increased expression of NKG2A in COVID-19 individuals (20). More importantly, in patients convalescing after therapy, the numbers of NK and CD8+ T cells were restored with reduced expression of NKG2A. In addition, these results suggested that the functional exhaustion of cytotoxic lymphocytes was directly associated with severity of SARS-CoV-2 infection. Hence, SARS-CoV-2 infection is likely to paralyze the antiviral immunity at an early stage and contribute to progression and severity of the disease (20) (Figure 1). In patients infected with SARS-CoV-2, NKG2A expression was increased significantly on NK and CD8+ T cells compared to those in healthy controls (20). Lower percentages of CD107a+ NK, IFN-γ+ NK, IL-2+ NK, and TNF-α+ NK cells and decreased mean fluorescence intensities (MFI) of granzyme B+ NK cells were also reported in COVID-19 individuals when compared to healthy controls (20). Although these preliminary results are of significance, they still would need to be reproduced by other laboratories, however, they clearly point to the potential contribution of functional exhaustion of cytotoxic lymphocytes in COVID-19 disease (20). Thus, future studies should be focused to fully understand the contribution of dysfunctional NK cells in disease pathogenesis. In addition, the underlying mechanisms of depletion of NK cells such as viral infection of NK cells, mobilization and homing of NK cells to the tissues from the peripheral blood, and activation induced cell death would need to be investigated (Figure 1).

**Figure 1:**
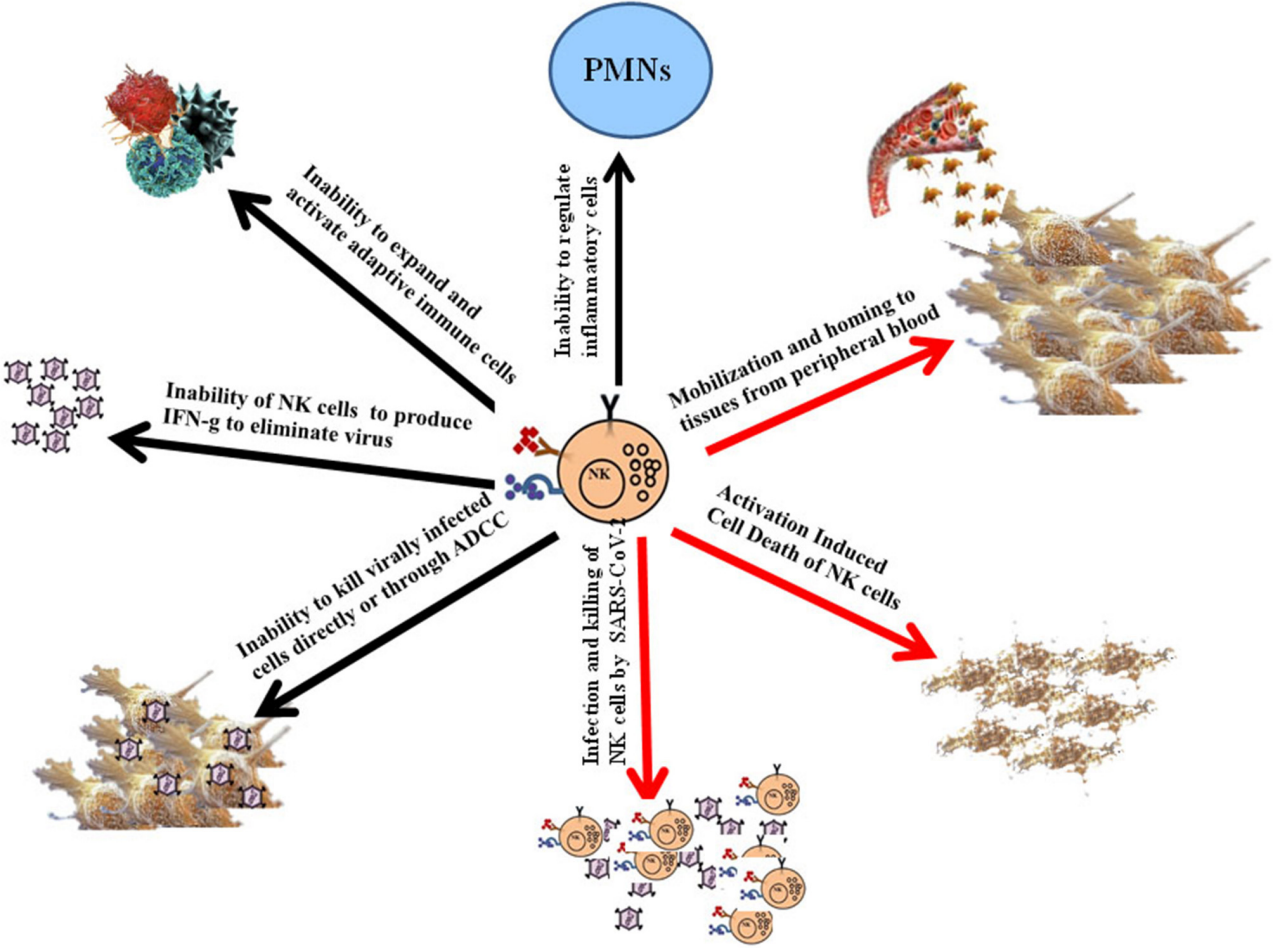
Schematic representations of potential mechanisms for the induction of dysfunction of NK cells, and the consequences of dysfunctional NK cells on pathogenesis of COVID-19 disease. Although the lack of NK cells in the peripheral blood of patients with COVID-19 disease could potentially in part be due to their mobilization and homing to different tissue compartments, it is more likely that these cells are adversely affected by the infection. Infection of NK cells by the virus and/or activation induced cell death of NK cells during infection could represent potential mechanisms of inactivation and depletion of NK cells in COVID-19 disease (Red Arrows). The consequences of such depletion and functional inactivation of NK cells in disease pathogenesis can be several fold as shown in the figure (Black Arrows).

Neutrophils are the most abundant white blood cells in the lung, and they are critical effectors against infections, in particular against bacterial infections, however they are also capable of inducing life-threatening morbidities. Moreover, Natural Killer cells are the most abundant lymphocytes in the lung, and therefore, play an important role not only in curtailment of infection but also in exertion of significant regulatory effect (21). However, little is known regarding the specific mechanisms by which NK cells maintain local homeostasis. By using lung-intravital microscopy to directly visualize and quantify neutrophil and natural killer cell interaction within the lung of live mice, the authors reported in a preliminary study that NK cells were greatly responsible for the slower pace of scanning of endothelium by neutrophils over a large area, and they were able to reduce the number of neutrophils that accumulated in an LPS-triggered inflammatory challenge (21). Indeed, depletion of NK cells in mice exhibited severe respiratory distress associated with protein-rich, high-permeability alveolar edema accompanied by neutrophil infiltration in myocardial infarction model (22). Thus, NK cells are important effectors in not only combating the infection directly but also indirectly by activating the local inflammatory processes to curtail infection. In addition, they are also able to limit local immune activation in the lung to a manageable level without causing or allowing significant pathologies to be induced by other immune effectors (Figure 1). Thus, NK cells are important in keeping the balance of immune activation in such a way that sufficient levels of activation will ensue to remove the infection in the presence of finely tuned inflammatory processes to avoid local tissue damage.

As mentioned above the infectious agent of COVID-19 disease depletes NK cells in the peripheral blood, and potentially even in the lung tissues of patients, thereby, disabling and depleting the core immune effectors necessary to remove the virus and regulate uncontrolled immune activation. Indeed, NK cells are the army generals of the immune effectors which bring order and discipline to the infected tissue microenvironment. Without the NK cells it is likely that immune anarchy may ensue and result in the uncontrolled expansion and activation of other immune effectors (16–19). We have previously shown that NK cells also curtail the numbers of CD4+ T cells and expand CD8+ T cells (23, 24) and (manuscript submitted). Therefore, lack of NK cells may also result in the decrease expansion of CD8+ T cells as seen in COVID-19 individuals (16–19) (Figure 1). Thus, it is no surprise that patients suffering from COVID-19 disease have greater CD4/CD8 ratios as reported previously.

The COVID-19 individuals suffer from increased viral replication as well as uncontrolled inflammation resulting in cytokine storm and widespread tissue and organ damage (25–27). NK cells are known to mediate cytotoxicity, and regulate both the innate and adaptive immune functions through the release of many pro- and anti-inflammatory growth factors, cytokines and chemokines (16–19, 28, 29). They constitute 5–15% of the peripheral blood mononuclear cells (PBMCs), and are cytotoxic effectors in the blood of healthy individuals with the ability to recognize and lyse virally infected cells, including SARS-CoV-2 infected cells and a number of different cancer stem cells (CSCs) and undifferentiated or poorly differentiated tumors which constitute the most aggressive subpopulations of the tumors. Morphologically, NK cells are large granular lymphocytes that develop in the bone marrow. After development, the majority of NK cells are found in peripheral blood as the third largest lymphocyte population, next to B and T cells (30). Moreover, NK cells are also found in the tissues such as in healthy skin, gut, lung, liver, lymphoid organs and uterus during pregnancy (31). NK cells have two different effector functions: cytotoxicity and cytokine release. In contrast to CD8+ cytotoxic T lymphocytes, NK cells do not need priming with antigen in order to kill their target cells. Their function is regulated by the sum of interactions between activating and inhibitory receptors on their surface and the ligands on the target cells (32). Ligands for NK activating receptors are expressed on fast proliferating cells that are virally infected or malignantly transformed. Cytotoxic activity of NK cells is executed by two distinct mechanisms. One is regulated by the cytotoxic granules containing perforin and granzymes. After the formation of the immune synapse between NK and target cells, cytotoxic granules are released. Perforin alters the permeability of the target cell membrane, allowing the entry of granzymes. The second pathway is interaction of ligands on NK cells with their respective cell death receptors on target cells. Target cell death can also be induced through antibody dependent cellular cytotoxicity (ADCC) (16–19). Indeed, NK cell mediated ADCC is likely one of the key mechanisms by which antibodies induced by the virus in recovered patients known as convalescent plasma or serum are able to alleviate the symptoms and improve the disease outcomes in infected and recovering patients (33) (Figure 1).

The second key effector function of NK cells is the release of cytokines and chemokines. Two major cytokines released by NK cells are IFN-γ and TNF-α (16–19, 34). Released cytokines not only affect the function of innate and adaptive immune cells, but it can also impact the differentiation of both healthy and cancer cells (Figure 1).

Similar to SARS-CoV-2 infection in patients, decreased NK cell function in the tumor microenvironment, and peripheral blood of cancer patients as well as down-modulation of CD16 receptors on the surface of NK cells have been reported previously (23, 35–40). Decreased function of NK cells is associated with increased viral infection and cancer risk, whereas higher function was correlated with prevention of establishment and progression of infection and cancer (16–19). Indeed, older patients and those with immunosuppression are more susceptible to severe form of SARS-CoV-2 infection, and are likely to die from it. Thus, it is no surprise that the same subsets of population of individuals are found to have lower expansion and functions of NK cells as reported previously (41, 42).

Decreased NK and T cell numbers and function can be due to the activation induced cell death and/or direct infection of the immune cells by the virus (Figure 1). Indeed, recent studies indicated that similar to MERS-CoV infection, SARS-CoV-2 also infects T cells through receptor-dependent, S-protein mediated membrane fusion and that the infection can be inhibited by EK1 peptide (43). Furthermore, the infection is abortive since SARS-CoV-2 does not have the capability to replicate in the T cells (43).

With promising studies demonstrating significant decreases and functional deficiencies in immune cell populations in particular the NK cells in COVID-19 patients, immunotherapy-based treatments using NK cells and CD8+ T cells and/or enhancement of the proliferation and function of NK cells in patients may present a significant and viable avenue toward mitigating disease establishment and progression.

## Data Availability Statement

The original contributions presented in the study are included in the article/supplementary material, further inquiries can be directed to the corresponding author/s.

## Author Contributions

AJ prepared the manuscript.

## Conflict of Interest

The author declares that the research was conducted in the absence of any commercial or financial relationships that could be construed as a potential conflict of interest.
